# Enhancement of early proximal caries annotations in radiographs: introducing the Diagnostic Insights for Radiographic Early-caries with micro-CT (ACTA-DIRECT) dataset

**DOI:** 10.1186/s12903-024-05076-x

**Published:** 2024-10-30

**Authors:** Ricardo E. Gonzalez Valenzuela, Pascal Mettes, Bruno G. Loos, Henk Marquering, Erwin Berkhout

**Affiliations:** 1 Department of Oral Radiology, Academic Centre for Dentistry Amsterdam, Universiteit Van Amsterdam and Vrije Universiteit Amsterdam, Amsterdam, Netherlands; 2 Department of Biomedical Engineering and Physics, Amsterdam University Medical Center, Universiteit Van Amsterdam, Amsterdam, Netherlands; 3 Video & Image Sense Lab - Informatics Institute, Universiteit Van Amsterdam, Amsterdam, Netherlands; 4 Department of Periodontology, Academic Centre for Dentistry Amsterdam, Universiteit Van Amsterdam and Vrije Universiteit Amsterdam, Amsterdam, Netherlands

**Keywords:** Dataset, Radiography, X-ray microtomography, Dental caries, Artificial intelligence, High-quality annotations

## Abstract

**Background:**

Proximal caries datasets for training artificial intelligence (AI) algorithms commonly include clinician-annotated radiographs. These conventional annotations are susceptible to observer variability, and early caries may be missed. Micro-computed tomography (CT), while not feasible in clinical applications, offers a more accurate imaging modality to support the creation of a reference-standard dataset for caries annotations. Herein, we present the Academic Center for Dentistry Amsterdam—Diagnostic Insights for Radiographic Early-caries with micro-CT (ACTA-DIRECT) dataset, which is the first dataset pairing dental radiographs and micro-CT scans to enable higher-quality annotations.

**Methods:**

The ACTA-DIRECT dataset encompasses 179 paired micro-CT scans and radiographs of early proximal carious teeth, along with three types of annotations: conventional annotations on radiographs, micro-CT-assisted annotations on radiographs, and micro-CT annotations (reference standard). Three dentists independently annotated proximal caries on radiographs, both with and without micro-CT assistance, enabling determinations of interobserver agreement and diagnostic accuracy. To establish a reference standard, one dental radiologist annotated all caries on the related micro-CT scans.

**Results:**

Micro-CT support improved interobserver agreement (Cohen’s Kappa), averaging 0.64 (95% confidence interval [CI]: 0.59–0.68) versus 0.46 (95% CI: 0.44–0.48) in its absence. Likewise, average sensitivity and specificity increased from 42% (95% CI: 34–51%) to 63% (95% CI: 54–71%) and from 92% (95% CI: 88–95%) to 95% (95% CI: 92–97%), respectively.

**Conclusion:**

The ACTA-DIRECT dataset offers high-quality images and annotations to support AI-based early caries diagnostics for training and validation. This study underscores the benefits of incorporating micro-CT scans in lesion assessments, providing enhanced precision and reliability.

**Graphical Abstract:**

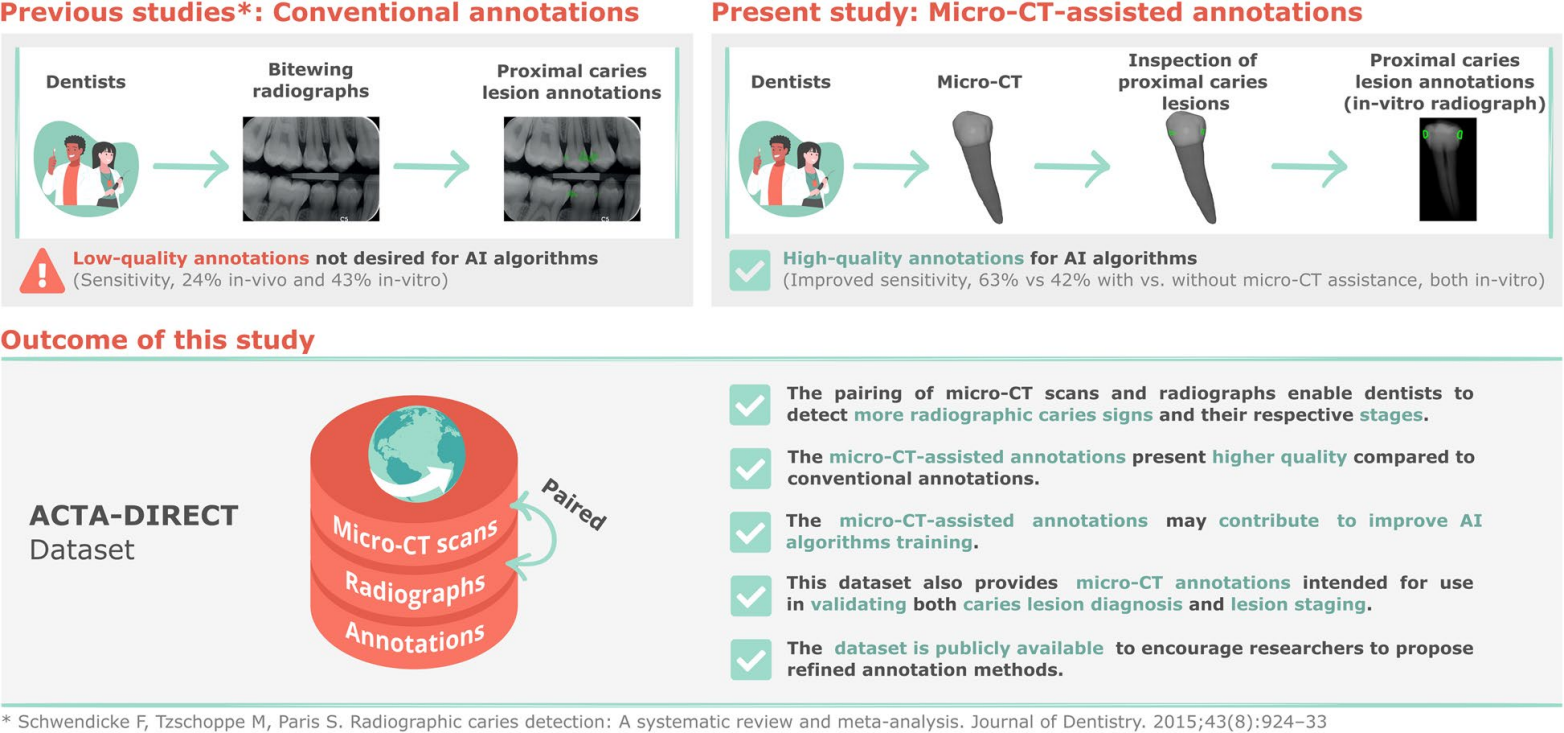

## Background

Dental caries (of tooth decay) are typically assessed in clinical settings through visual inspections. Radiographs, particularly bitewing views, are also advisable as ancillary support and are especially useful in detecting proximal caries [1]. Proximal caries are defined here as early caries, when confined to enamel layer or outer third of the dentine. Facilitating their discovery may help preserve tooth structure and prevent invasive treatment [2]. A study has shown that when proximal radiolucency was confined to the enamel in most of the cases (87%) there was no clinical cavitation present [3].

A meta-analysis in which proximal lesions were assessed by inspecting conventional radiographs has disclosed sensitivities of only 24% in vivo and 43% in vitro [4]. Moreover, it is generally presumed that many early lesions, restricted to outer enamel, remain undetected [5]. Although a number of researchers have applied artificial intelligence (AI) algorithms to aid in this process, they still have their own limitations [6–9]. Due to the inherent challenges in detecting proximal caries lesions, the AI system performance may be impacted [10]. A high-quality reference standard is required for training and validating AI algorithms [6].

Prados-Privado et al. have advocated that a histologic reference standard be used for this purpose [6]. However, stains and microbial byproducts may be misinterpreted as carious lesions in histologic preparations [11]. Furthermore, extrapolating input from histologic sections to radiographic images also involves subjective interpretations [11, 12].

Micro-CT holds promise as a viable alternative to histology, offering a reference standard for annotation of caries [11, 13, 14]. Unlike a histologic approach, better sensitivity and accuracy have been achieved with regard to enamel and dentine lesions [13]. This edge may not only enhance the accuracy of radiographic annotations, but also pave the way for greater precision in AI training, especially when identifying early-stage caries.

The core issue driving our investigational efforts is whether micro-CT imaging significantly enhances the reliability of radiographically annotated early proximal carious lesions, generating reference-standard data. We have therefore constructed the ACTA—Diagnostic Insights for Radiographic Early-caries with micro-CT (ACTA-DIRECT) dataset, aiming to establish a new benchmark for accurate identification of early-stage proximal caries. This dataset is an open-access resource, offering a micro-CT-based reference standard to support more accurate early proximal caries annotations in radiographs. Our objective was to construct a dataset designed to facilitate AI algorithm training and validation through the provision of micro-CT-assisted radiographic annotations, thereby dispensing with the need for micro-CT involvement beyond this early stage.

## Methods

**Fig. 1:**
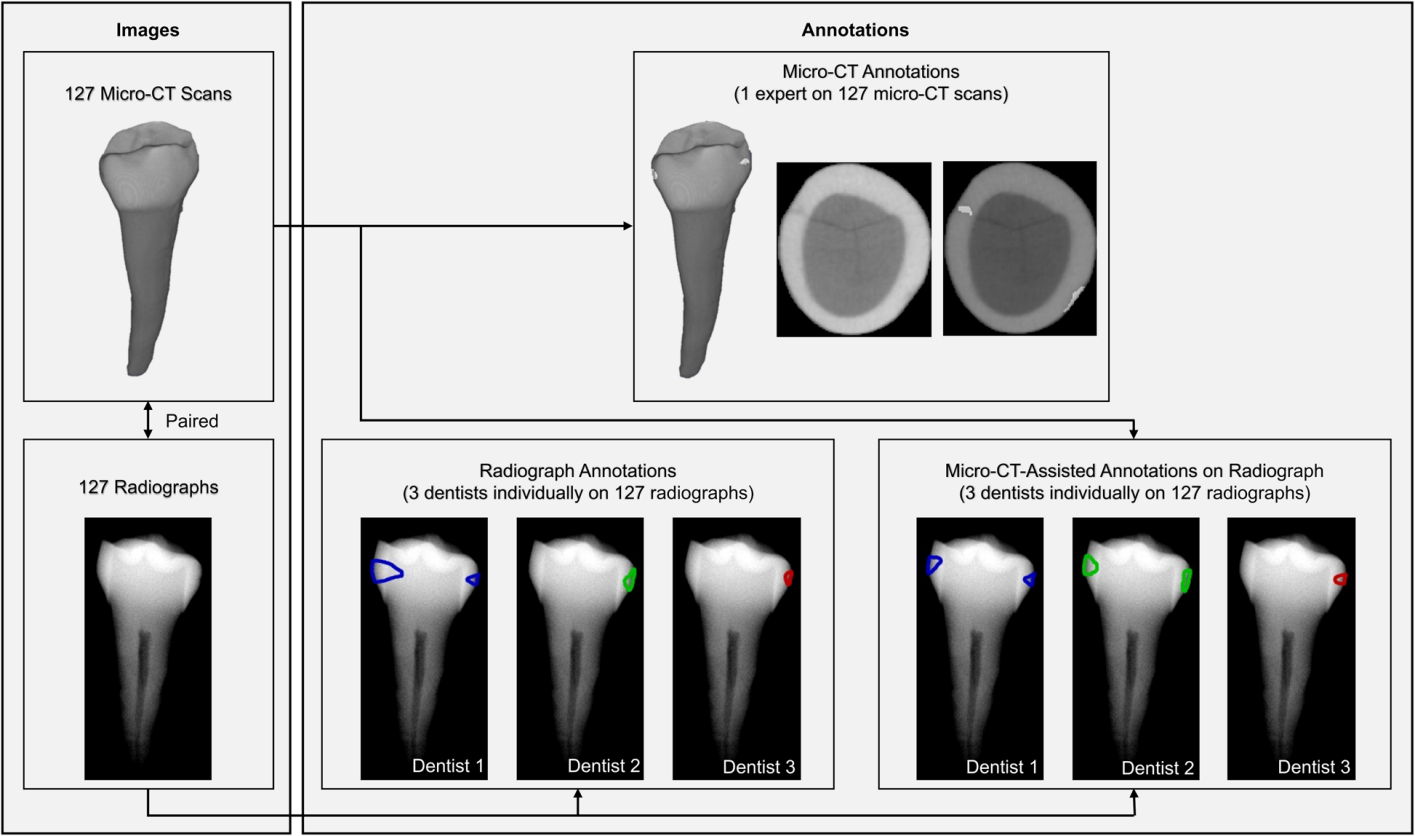
Structure of the dataset used for caries stage analysis, including 127 micro-CT scans and corresponding radiographs, with annotations provided by one expert on micro-CT scans and three dentists on radiographs. The annotations, originally binary masks (0 = background, 1 = caries), are shown as delineations for clarity

The aim of this study is to construct a paired dataset of micro-CT scans and radiographs, and to provide an annotation method that integrates the analysis of micro-CT scans with radiographic inspections, in order to capitalize on the benefits of micro-CT for identifying proximal early caries lesions in radiographic images, see Fig. 1.

### Tooth imaging

In our study, we selected 179 extracted tooth samples from a large collection maintained for educational purposes in the ACTA Radiology Department. The samples were preserved in sodium hypochlorite (solution 2% buffered), which was diluted in water in a ratio of 4 parts water to 1 part solution, an adequate solution for maintaining their integrity. The inclusion criteria were molar and premolar teeth that were either sound or exhibited early proximal caries lesions. The exclusion criteria entail excluding any teeth with dental restorations.

We acquired micro-CT scans of individual teeth using a micro-CT scanner (MicroCT40; SCANCO Medical AG, Brüttisellen, Switzerland) at the following settings: tube voltage, 70 kV; tube current, 114 μA; field of view (FOV), 36.9 mm; and voxel size, 72 μm. Standard radiographs were then taken of each tooth positioned with the buccal side facing the tube of the Planmeca machine. We used a Planmeca INTRA (Planmeca Oy, Helsinki, Finland) unit, equipped with a long tube. The exposure time was adjusted to 0.32 s to accommodate the extended focus-detector distance, along with a tube voltage of 60 kV and a tube current of 8 mA. A Planmeca Prosensor CCD-sensor was used for capturing the radiographs. The spatial resolution of the CCD-sensor is 17 lp/mm. The micro-CT images were acquired with a 16-bit depth, while the radiographs were captured at 12-bit depth.

**Fig. 2:**
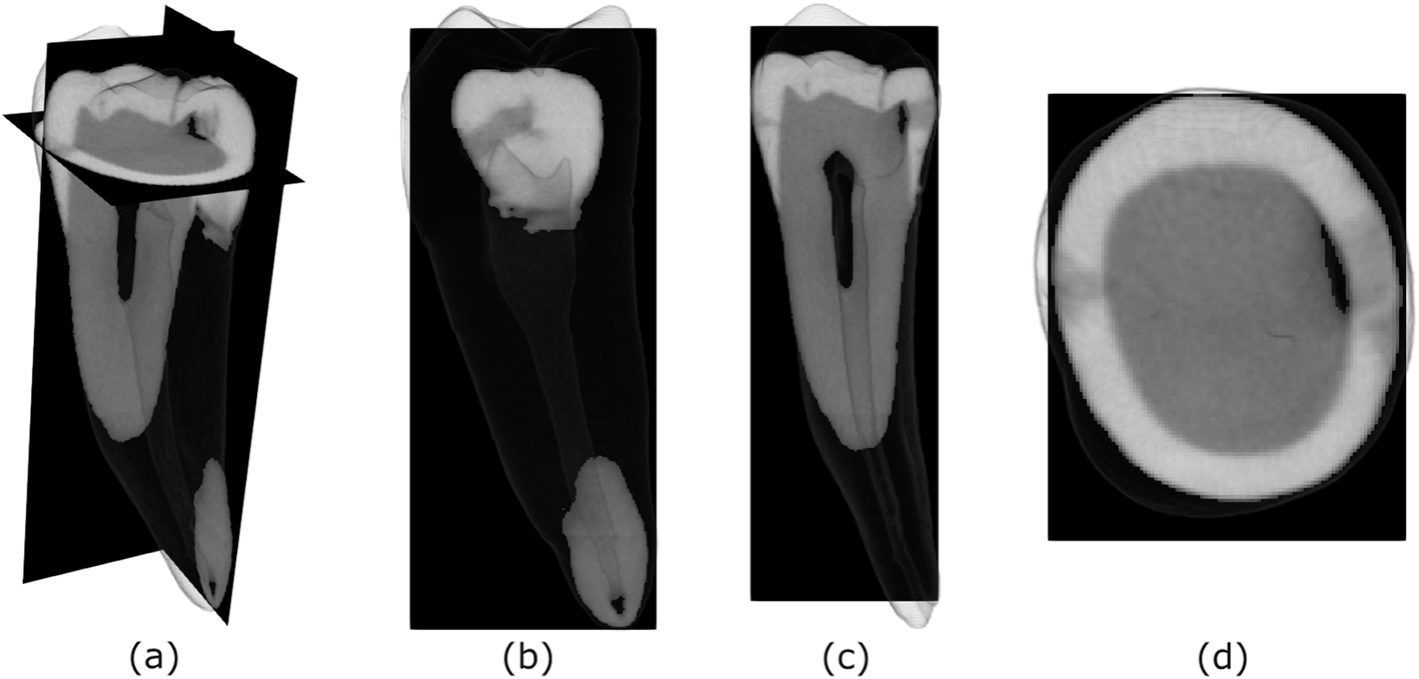
Micro-CT scan views: **a** three-dimensional, **b** coronal, **c** sagittal, and **d** axial views

Figure 2 shows a micro-CT scan of tooth with two proximal caries: Locations/depths of proximal lesions discernible in structurally transparent. In each plan view, one slice allowing the clear observation of the caries lesion was selected.

**Fig. 3:**
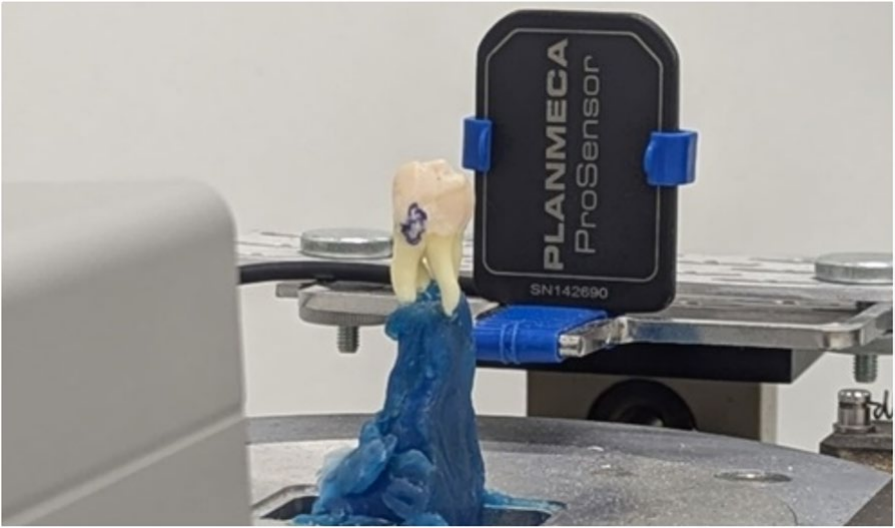
Setup for acquiring individual tooth radiographs

In order to acquire the standard radiographs, each tooth was individually positioned on a specially designed rotatable plateau to ensure consistent orientation. The teeth were stabilized using dental wax, which functioned as a mold, securing the teeth in a fixed position. The focus-to-object distance was standardized at 42.5 cm, with the object-to-sensor distance maintained at 6.5 cm. The sensor was mounted in a holder to achieve stability, see Fig. 3.

This body of information was dubbed the ACTA-DIRECT dataset. Ultimately, it was to be made available as an open-source research tool.

### Annotations

A freeware application (3D Slicer 5.0.2; www.slicer.org) facilitated inspection and manual segmentation of proximal caries [15]. The manually segmented caries lesions, herein referred to as ‘annotations’, are critical for the subsequent analysis and interpretation of lesion characteristics. Three qualified dentists, with between 5 and 25 years of experience, performed all radiographic annotations. Each was instructed to address only proximal caries, excluding those at cementoenamel junction. A calibration session was conducted beforehand in a dimly lit room. Importantly, no dataset images were used for calibration purposes. We used the SMPTE test pattern for quality control of the monitors. Annotators were allowed to adjust brightness and contrast in 3D Slicer for better visualization.

In subsequent scoring sessions, these dentists were required to independently annotate proximal carious lesions in each radiograph. All sessions similarly were performed in dim background lighting to minimize glare and distractions. Screen settings were calibrated to achieve optimal contrast resolution. Two annotation strategies were undertaken separately, 2 weeks apart. Both conventional (no input from micro-CT scans) and micro-CT-assisted (corresponding micro-CT scans referenced) annotations were carried out. In the case of the micro-CT-assisted annotations, each dentist used two displays: one to analyze the micro-CT scan and the other to annotate the caries lesion in the radiograph. To improve analysis, the dentists could align the 3D scan to match the 2D radiograph, making it easier to locate and annotate the caries lesion in the radiograph. The complete micro-CT files, without any cropping, were provided to the dentists, allowing them to analyze every slice and from every direction.

A dental radiologist, with 25 years of experience in clinical dentistry and oral radiology, independently annotated all caries visible in micro-CT tooth scans. The definition of 'caries lesion' and the selection of thresholds were primarily based on the radiologist's expertise, supported by the software's recommendations. This rigorous process involved meticulously examining individual slices in all planes (coronal, axial, and sagittal) and annotating proximal caries lesions one slice at a time. Additionally, the expert had direct access to inspect the extracted teeth to further refine the annotations. To ensure accuracy and consistency, all annotations were later cross-referenced with those produced by the other dentists in radiographs. These micro-CT annotations were to be utilized as reference standards. Note that the annotations made by the expert on the micro-CT slices were not provided for use during the micro-CT-assisted annotations of radiographs, only the original micro-CT files were provided.

**Fig. 4:**
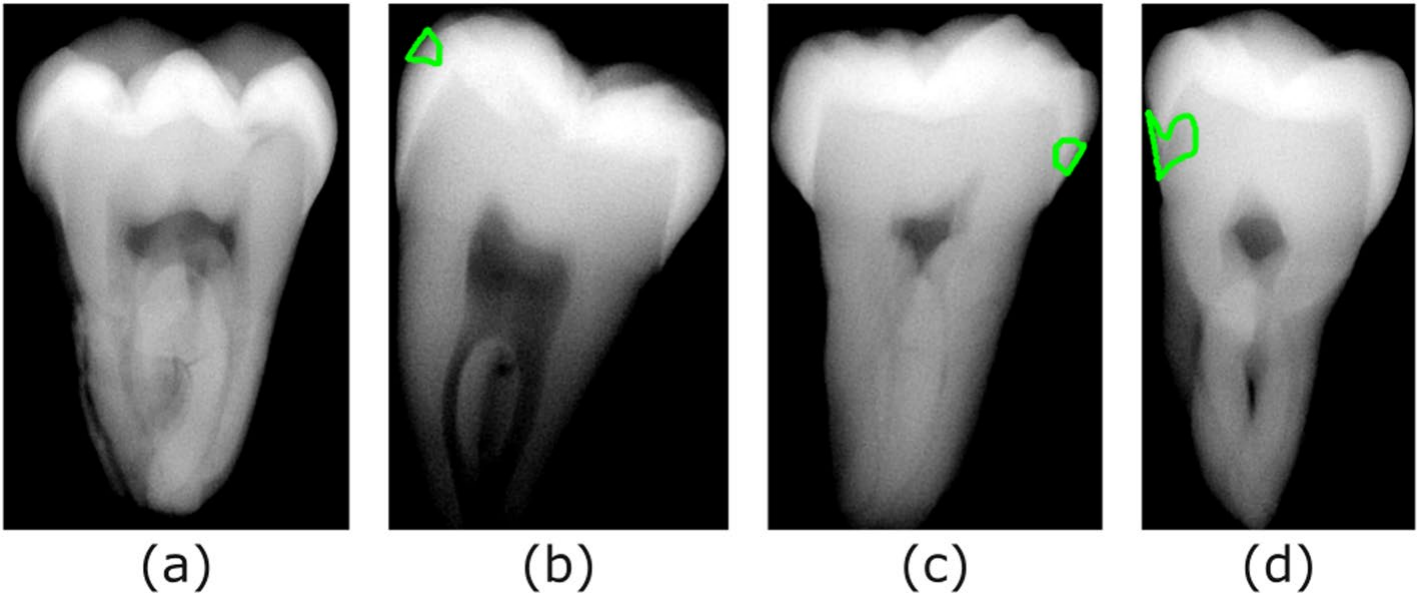
In-vitro radiographs showing caries lesion annotations. The corresponding perceived caries lesion stages are: **a** Sound, **b** RA1, **c** RA2, **d** RA3

Altogether, 179 teeth were subject to assessment and annotation, for a total of 358 proximal surfaces. We categorized each lesion by applying the International Caries Classification and Management System (ICCMS) scoring protocol of the International Caries Detection and Assessment System (ICDAS) Foundation [16]. However, our dataset was restricted to absence of or early-stage caries, stipulated in the ICCMS as follows: (1) sound teeth (no caries, Fig. 4a), (2) radiolucency in outer half of enamel (RA1, Fig. 4b), (3) radiolucency in inner half of enamel ± enamel-dentin junction (RA2, Fig. 4c), or (4) radiolucency confined to outer third of dentin, immediately underlying enamel (RA3, Fig. 4d). All annotations on the micro-CT and radiographs were classified according to ICMMS scoring protocol by an expert to assign the corresponding caries lesion stage.

### Statistical analysis

To compare conventional and micro-CT-assisted annotation methods, we assessed both interobserver agreement and diagnostic accuracy. Interobserver agreement entailed the following calculations: (1) average Cohen’s Kappa for each observer pair; (2) 95% confidence intervals (CIs) using standard error of Cohen's Kappa values. In terms of diagnostic accuracy, calculations were as follows: (1) average performance metrics (sensitivity, specificity, balanced accuracy, positive predictive value [PPV], and negative predictive value [NPV]) across dentist annotations; (2) 95% CIs using Wilson Score method for binomial proportion. Additionally, to compare the agreement between the caries lesion stages assigned to each annotation in radiographic and micro-CT images, we calculated the linear weighted Kappa value for each dentist.

For an overall view of diagnostic accuracy demonstrated by both methods, we generated respective receiver operating characteristic (ROC) curves and determined area under the curve (AUC) values, with 95% CIs computed by *t*-distribution method.

## Results

Table 1 shows the dataset distribution of ICCM categories, based on reference-standard annotations. Most of the proximal sides were sound, only 36% displaying caries in early stages of development (RA1, 17%; RA2, 18%; RA3, 1%).

**Table 1:** Distribution of proximal surfaces with/without early caries (based on ICMMS) in ACTA-DIRECT dataset

ICCM caries stage	Surface count (%) (*n* = 358)
Sound	228 (64)
RA1	59 (17)
RA2	66 (18)
RA3	5 (1)

Table 2 presents the weighted Kappa values, which measure the agreement between the caries lesion stages indicated in radiographic annotations by each dentist and the corresponding caries lesion stages indicated in the Micro-CT annotations. The comparison is made for both the conventional radiograph annotation method and the Micro-CT-assisted annotation method. Higher Kappa values suggest a stronger agreement with the Micro-CT ground truth, indicating that the annotations made using the Micro-CT-assisted method are more consistent with the Micro-CT data compared to the conventional method.

**Table 2:** Weighted Kappa values comparing caries lesion stage (ICCMS) indicated for micro-CT annotations and radiographic annotations

Annotator	Conventional Annotation	Micro-CT-assisted annotation
Dentist 1	0.36	0.57
Dentist 2	0.35	0.67
Dentist 3	0.43	0.50

Interobserver agreement levels for conventional and micro-CT-assisted annotation attempts were 0.46 (95% CI: 0.44–0.48; moderate agreement) and 0.64 (95% CI: 0.59–0.68; substantial agreement), respectively. Thus, micro-CT assistance readily surpassed a conventional approach.

Performance metrics (i.e., sensitivity, specificity, PPV, and NPV) of the three dentists are listed in Table 3 by annotation method. Micro-CT assistance regularly improved all metrics, compared with conventional annotation. Specifically, average sensitivity climbed from 42% (CI: 34–51%) to 63% (CI: 54%-71%), with average specificity rising modestly from 92% (CI: 88–95%) to 95% (CI: 92–97%). Notable improvements in balanced accuracy (from 67 to 79%), PPV (from 75 to 88%), and NPV (from 74 to 82%) were also evident, underscoring the effectiveness of micro-CT assistance in enhancing diagnostic accuracy during dental assessments.

**Table 3:** Dentists performance metrics for proximal caries detection, shown by annotation method

Annotator	Annotation method	Sensitivity	Specificity	Balanced accuracy	PPV^a^	NPV^b^
Dentist 1	Conventional	48 (39–56)	88 (83–92)	68 (63–73)	70 (59–78)	75(69–80)
	Micro-CT-assisted	67 (58–74)	93 (89–96)	80 (75–84)	84 (76–90)	83 (78–87)
Dentist 2	Conventional	27 (20–35)	98 (96–99)	63 (57–67)	90 (76–96)	70 (65–75)
	Micro-CT-assisted	69 (61–77)	98 (95–99)	84 (79–87)	95 (87–98)	85 (80–89)
Dentist 3	Conventional	52 (43–60)	89 (84–92)	70 (65–75)	73 (63–81)	76 (71–81)
	Micro-CT-assisted	52 (44–61)	95 (91–97)	74 (69–78)	85 (76–91)	78 (72–82)
Average	Conventional	42 (34–51)	92 (88–95)	67 (62–72)	75 (64–83)	74 (68–78)
	Micro-CT-assisted	63 (54–71)	95 (92–97)	79 (74–83)	88 (80–93)	82 (77–86)

All data expressed as % (95% confidence interval)

^a^*PPV *Positive predictive value

^b^*NPV* Negative predictive value

Diagnostic accuracy was higher overall for micro-CT-assisted annotation; and on an observer level, it was apparent that annotation accuracy benefits from referencing of micro-CT scans. In particular, the degree of sensitivity increased substantially.

**Fig. 5:**
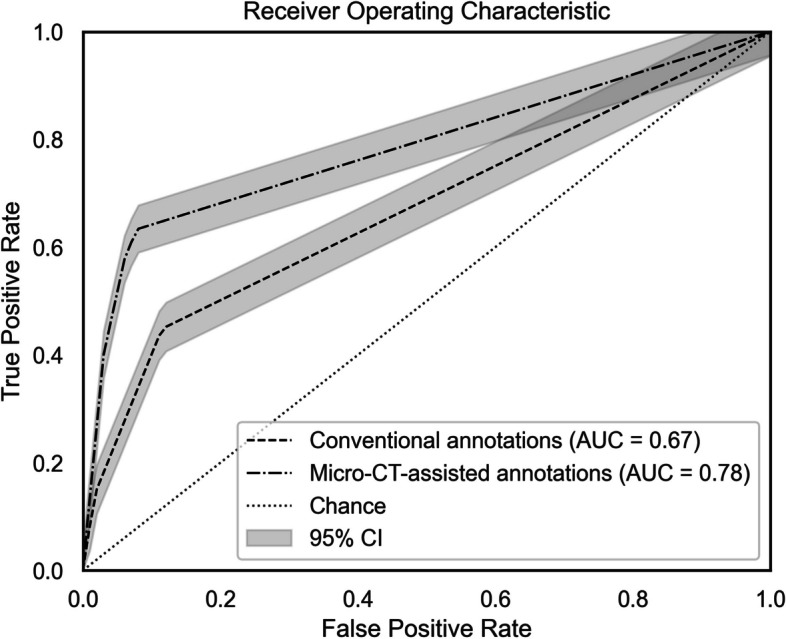
ROC curves for average diagnostic performances of annotation methods

Receiver operating characteristic (ROC) curves plotted in Fig. 5 show how well caries were detected by both methods. The micro-CT-assisted approach (area under the curve [AUC] = 0.78) outperformed conventional annotation (AUC = 0.67) considerably.

## Discussion

The present study highlights the ACTA-DIRECT dataset of radiographs and micro-CT dental scans. All proximal tooth surfaces were either sound or presented with early caries. The value of the ACTA-DIRECT dataset is primarily derived from its integration of micro-CT and radiograph images, coupled with high-quality lesion annotations, setting it apart as a singular resource in scientific research.

The ACTA-DIRECT dataset carves out a unique niche in ongoing research by focusing on early caries, a topic often neglected in traditional research. What sets our work apart is not the diagnostic standard set by micro-CT, but rather its pioneering implementation for radiographic annotation assistance. This approach addresses a critical gap in the field. The traditional approach relying on radiographs, has inherent limitations (such as low sensitivity) that may bias subsequent annotations [4]. Such inaccuracies will likely hamper development of reliable AI algorithms trained to detect carious lesions [9]. Micro-CT-assisted radiographic annotations are simply more compelling and pave the way for training of AI algorithms with greater precision, especially in detecting early-stage caries. We believe that caries are best diagnosed in early stages still be treated with preventive measures. The difficulty dentists face is exemplified by the higher sensitivity of bitewing X-rays for lesions with (versus without) cavitation, contributing to poorer diagnostic success and lesion progression [17]. The ACTA-DIRECT dataset is a valuable resource for research on automated or AI-based lesion detection, aiming to improve caries diagnosis.

The ACTA-DIRECT dataset was meticulously constructed. Nonetheless, we must acknowledge certain intrinsic limitations. First, we did not assess the reproducibility of the reference standard. Yet, as explained earlier we applied multiple measures to ensure that this reference standard is accurate and independent of the rater. Second, incompatibility between 3D micro-CT annotations and 2D clinical annotations is another drawback that may prove challenging for clinical integration. Disparities in dimensional representations may cause misinterpretations or information loss upon transitioning. To mitigate this problem, we have formulated comprehensive guidelines and training for dentists engaged in micro-CT-assisted annotations to minimize subjectivity and boost annotation consistency. Indeed, our statistical analysis has documented a significant difference in interobserver agreement when comparing methods, attesting to the enhanced consistency and reliability of a micro-CT-assisted approach. Additionally, the weighted Kappa values showed greater agreement between the caries lesion stages in the micro-CT-assisted annotations and the micro-CT annotations, compared to the conventional method, further highlighting the advantage of the micro-CT-assisted approach for producing more accurate annotations. Third, we acknowledge the imbalance in caries stage presence and depth as outlined in Table 1 compared to the general population. We aimed to generate a dataset designed to efficiently train and evaluate AI models for the detection of early proximal caries lesions and the inclusion of sound surfaces. This requires to include more teeth with lesions than can be found in the general population. On the one hand, by enhancing the utility of AI by focusing on early caries lesions, the generalizability of our findings may be reduced. On the other hand, advanced caries are typically easier to detect, due to visual and tactile properties of cavities [18]. Fourth, although one of the goals of creating this dataset is to improve early caries detection and encourage preventive treatment, it is known that detecting carious lesions at any stage can sometimes lead dentists to opt for invasive treatments. This could counteract the intended preventive benefits. Such issues could be mitigated through targeted recommendations and improved education. Fifth, we recognize a fundamental limitation in that the 2D radiographs used in our study are not directly translatable to clinical bitewing radiographs. This limitation could be addressed by initially training AI models with controlled data and subsequently refine them using real-world data to ensure generalizability, which has been recognized as a balanced approach to pilot AI algorithms [19]. Sixth, the CCD detectors were chosen for their ability to produce clear and high-contrast images, which were necessary for the data acquisition process. As this study was not intended to be generalized to clinical practice, comparing different radiographic technologies was beyond its scope.

Our results confirm the superior reliability of the micro-CT-assisted method for proximal caries annotation compared to the conventional method. In terms of accuracy, micro-CT assistance outperformed a conventional approach, achieving significant improvement in performance metrics.

The high diagnostic accuracy of micro-CT (versus traditional radiography) has innate benefits [13]. Its high resolution and 3D depictions resolve issues common in 2D radiographs, allowing thorough scrutiny of suspected caries. The scanning resolution we applied to detect focal demineralization (dataset included) was 72 µm. This is not the highest achievable resolution on the used scanner, but higher resolution would merely prolong scanning times and require more storage space, adding little or no information [14]. We acknowledge that reference standards should aim for maximum detail, however we determined that caries lesions are sufficiently detectable at a 72 µm resolution. Therefore, we deliberately chose this resolution as comparable resolutions may be obtained with cone-beam CT (CBCT) scanners, which is another option for high-resolution scanning of extracted teeth. One might consider CBCT use to clinically generate reference standards for caries research. However, surrounding anatomic structures and artifacts (caused by neighboring teeth and restorations) are apt to impair resolution and detectability of small caries, compared with in vitro scans. Furthermore, there are ethical concerns raised by delivery of relatively high-dose radiation to acquire reference standards.

The high quality of our in vitro ACTA-DIRECT dataset also has a downside, namely a gap in clinical application. Even with micro-CT support, the average sensitivity recorded for annotations in radiographs only reached 63%. Consequently, other methods are needed to render data from micro-CT scans suitable for broader clinical research applications and training AI algorithms. Given the substantial progress anticipated for AI algorithms in automated dental image interpretation, greater accuracy in diagnosis is expected [20]. The ACTA-DIRECT dataset may represent an important development in improving data quality. We expect the provided dataset to facilitate ongoing research and open avenues for improving diagnostic tools and clinical practices in dental healthcare. As part of our future work, we are committed to continuously expanding this dataset, and leverage it for AI models training.

Finally, although micro-CT-assisted annotation outperformed the conventional approach in detecting caries (Fig. 5), neither reached a perfect AUC of 1. It is worth noting that during micro-CT-assisted annotation, dentists had access to the micro-CT scans but not to the reference standard lesion annotations. In addition, the micro-CT-assisted radiographic annotations were not validated by additional dentists, unlike the procedure for reference standard annotations. Potential innovations, such as better imaging technology, refined annotation processes, or advanced algorithms, may be influential. Attaining a closer-to-perfect AUC should ensure more accurate caries detection.

## Conclusion

We have introduced the ACTA-DIRECT dataset, which is now publicly available as an open resource. This in vitro dataset includes micro-CT scans of 179 teeth, with corresponding radiographs. All proximal early caries identified in micro-CT scans are annotated, available as separate files. We have shown that with micro-CT support, the robustness and accuracy of annotating carious lesions are improved. Similarly, there is added value as a reference standard for training and validation of AI algorithms.

## Data Availability

The dataset generated during the current study is available in the VU YODA repository, 10.48338/VU01-WK8SQN.

## Abbreviations

ACTA: Academisch Centrum Tandheelkunde Amsterdam (Academic Centre for Dentistry Amsterdam)
AI: Artificial Inteliggence
AUC: Area Under the Curve
CI: Confidence Interval
CT: Computed Tomography
DIRECT: Diagnostic Insights for Radiographic Early-caries with micro-CT
FOV: Field of View
ICCMS: International Caries Classification and Management System
ICDAS: International Caries Detection and Assessment System
IRB: Institutional Review Board
NPV: Negative Predictive Values
ROC: Receiver Operating Characteristic
PPV: Negative Predictive Values
